# Gender linked fate explains lower legal abortion support among white married women

**DOI:** 10.1371/journal.pone.0223271

**Published:** 2019-10-10

**Authors:** Leah Ruppanner, Gosia Mikołajczak, Kelsy Kretschmer, Christopher T. Stout

**Affiliations:** 1 School of Social and Political Sciences, University of Melbourne, Melbourne, Victoria, Australia; 2 School of Public Policy, Oregon State University, Corvallis, Oregon, United States of America; University of California-Irvine, UNITED STATES

## Abstract

Abortion is uniquely connected to women’s experiences yet women’s attitudes towards legal abortion vary across the pro-choice/anti-abortion spectrum. Existing research has focused on sociodemographic characteristics to explain women’s levels of abortion support. Here, we argue that abortion attitudes vary with women’s perceptions of gender linked fate, or the extent to which some women see their fates as tied to other women. Drawing upon existing research showing that married white women report lower levels of gender linked fate than their non-married counterparts, we assess these relationships for abortion attitudes applying the 2012 American National Election Survey (*n* = 2,173). Using mediation analysis, we show that lower levels of gender linked fate among married white women (vs. non-married white women) explain their stronger opposition to abortion. As many state governments are increasingly legislating restricted access to legal abortion, understanding factors explaining opposition to legal abortion is urgently important.

## Background

On May 16, 2019, Alabama’s state governor signed the most restrictive abortion law to date, outlawing virtually all abortions (with risk to pregnant woman’s life being the only admissible exception) and altering penal sanctions such that medical doctors who perform abortions risk up to 99 years in prison. Five other U.S. states–Georgia, Kentucky, Missouri, Mississippi and Ohio–have also legislated heartbeat bills, or a ban of any abortion once a heartbeat of a fetus can be detected (i.e., 6 to 8 weeks after conception). The passage of these bills aims to push abortion legislation to the Supreme Court to overturn legal precedent. Legal abortion has historically been one of the most divisive political issues in American politics but the stakes for abortion access are amplified in the current political environment.

Abortion is often used as a litmus test for dividing conservative from liberal perspectives. Since 1973, when the Supreme Court legalized abortion across the United States with its *Roe v*. *Wade* decision, the issue has only grown in importance, as activists, doctors, and politicians have struggled over proposed and enacted state restrictions to abortion services. The political and moral debates around women’s access to safe and legal abortion have informed a broad research agenda seeking to understand the demographics of American opinion on reproductive rights, and abortion specifically. Here, we show that weaker perceptions of gender linked fate among married white women (compared to nonmarried white women) play a crucial role in explaining abortion attitudes.

Following *Roe v*. *Wade*, researchers were primarily interested in understanding cleavages in legal abortion support by race and education, with a surge in research in the 1980s (see e.g., [1, 2, 3, 4, 5, 6]). The results showed strong racial differences, with white women supporting legal abortion at higher rates than black women, even though the latter were three times more likely on average to have an abortion [1, 3, 7, 8]. Since the 1980s, however, racial gaps in support for legal abortion have narrowed, while other demographic determinants are growing in influence [8, 9, 10, 11, 12, 13]. College-educated women are historically more likely to support legal abortion [1, 12–15]. Those with higher incomes are also more likely to support legal abortion, underscoring that abortion support, along with other progressive attitudes, is tied to higher earnings [1, 4, 16, 17].

Conversely, religiosity is an important determinant of opposition to abortion, serving as a strong voting block issue. Evangelical Protestants are the most likely to oppose abortion, and are among the strongest advocates for legislation to nullify *Roe v*. *Wade* [4, 18, 19, 20, 21, 22, 23]. Similarly, recent research documents that constituents of Southern states are comprehensively less supportive of abortion than those in the Northern states [24, 25, 26]. The Southern states exhibit intense concentrations of conservative religiosity, and for many lawmakers hailing from the South, anti-abortion attitudes serve as important evidence of their moral conservatism [27, 28, 29]. Thus, it is no surprise that the Southern states–Missouri, Georgia, Kentucky and Mississippi–clustered in their passage of restrictive abortion laws in 2019 [30]. Here, we show that perceptions of gender linked fate–or seeing one’s future as linked to other women–helps explain support for legal abortion, extending existing research linking gender linked fate to conservative political attitudes [31].

Complicating this research, however, is the apparent lack of a gender effect for abortion support. As noted by Hout [32], women are more likely than men to occupy both extremes (pro-choice and anti-abortion) on the abortion spectrum. Although abortion is intrinsically tied to women’s bodies, past research has generally found that women are no more likely to support legal abortion than men, despite the fact that restricting abortion access disproportionately affects women [33, 34, 35]. The absence of gender differences has led some researchers to conclude that gender matters less than religious, educational and racial differences [36]. A substantial body of research has attempted to explain why women fail to support legal abortion at higher rates than men, with many attributing it to women’s reticence towards undergoing an abortion themselves, as well as how these attitudes vary based on circumstances that lead women to seek an abortion (for a review, see [36]). While others have focused on the Rossi Scale as a measure of abortion support, we use a single item measure of abortion support which identifies the intensity of support rather than variance in support by specific condition, which may not be ordinal or continuous [37]. In this regard, abortion is often understood from an individualized lens but may also capture variation in perceptions of a woman’s connection to other women. Here, we contribute to this conversation by assessing how differences in perceptions of gender linked fate explain differences in women’s abortion attitudes, depending on their marital status.

Gender linked fate measures the extent to which women perceive their futures to be tied to those of other women [38]. Individuals high in gender linked fate perceive their future resources and experiences to be tied to those who share the same gender. In the case of abortion, women’s futures are truly linked–regardless of one’s own likelihood of having an abortion, voting to reduce access to legal abortion will inherently affect the future lives of other women. Emerging research shows that gender linked fate varies by race and marital status with married white and Latina women reporting lower levels of gender linked fate than their non-married counterparts and, as a consequence, voting more conservatively [31]. These patterns may extend to abortion attitudes as well with weaker gender linked fate explaining lower support for legal abortion. We note here that our analyses do not replace other mechanisms that structure abortion attitudes–education, income, regionality, ideology and religion–but rather offer an additional theoretical mechanism to explain differences in women’s abortion attitudes.

As indicated above, we anticipate that the relationship between gender linked fate and abortion attitudes will vary by race. White and Latina conservative women are typically the most vocal opponents of abortion [19, 39] and white women and Latina women report lower levels of gender linked fate than black women [31]. Black women’s reproductive decisions are structured around a shared perception of racial inequity, structural inequality, poor life-chances, high rates of poverty and exposure to violence [40]. These factors tend to shape the African American community’s sense of ‘linked fate’ including their support for legal abortion [17, 38, 41]. Among black women, marital status is less important than race in binding women together along perceptions of gender linked fate [31]. As a consequence, black women may report higher levels of gender linked fate and support for legal abortion across all racial groups and marital statuses. In addition, black women’s support of legal abortion is net of their higher religiosity suggesting abortion attitudes are more complex than simple racial, marital or religious divides, tapping into the intersection of these identities. We explore these gaps paying careful attention to race and marital status and differences in gender linked fate.

We test our assumptions by utilizing nationally representative data from the 2012 American National Election Survey (ANES; n = 2,173) that measured gender linked fate, legal abortion support, marital status, and race. By employing mediation analysis, we identify both the direct and indirect impact of marital status on attitudes towards legal abortion. Specifically, we show that, among white women, differences in gender linked fate by marital status explain opposition to legal abortion net of other sociodemographic controls. These results are politically and socially important, given that white women are an increasingly influential demographic in American politics and the most conservative on abortion [39, 42]. Our results are especially relevant for restrictive abortion laws in states like Georgia and Alabama which have some of the highest concentrations of married white women in the nation [43]. Thus, the demographic concentration of married white female voters in certain states may help explain shifts in the political climate on hot-button issues like abortion, with gender linked fate an important explanatory mechanism to understand this changing political landscape.

### Group consciousness and gender linked fate

Existing research has largely focused on gender group consciousness but less is known about what structures perceptions of gender linked fate [44]. Group consciousness and gender linked fate, although interrelated, are distinct theoretical concepts [45, 46]. Group consciousness measures a person’s internalized group identity, along with their political awareness of their own gender, racial or class group’s position in society [44]. Linked fate captures the perception that an individual’s life chances are intrinsically tied to those who belong to the same demographic group. Strong group consciousness is often a precursor to developing a sense of linked fate but individuals can have group consciousness without linked fate [45, 47]. For example, a woman may see women as a subjugated group in society but may not see her personal life chances as tied to other women (i.e. group consciousness but no gender linked fate). Thus, those with a high level of group consciousness do not necessarily perceive their fate as linked to others in their demographic group [45]. Moreover, Sanchez and Vargas [46] demonstrate that group consciousness and linked fate have different consequences for behaviors across racial groups. Thus, we would expect that gender group consciousness may shape attitudes differently than gender linked fate with important between-group differences.

Since abortion is an issue that specifically affects women, gender linked fate may be an important mechanism to explain legal abortion support. To date, no study has established this link, an omission that likely reflects a dearth in data on gender linked fate as it is rarely included in large scale representative surveys. This omission is surprising given that racial linked fate is consistently shown to structure political attitudes and voting [38, 46, 47]. Fortunately, the 2012 American National Election Survey included gender linked as a one-off survey item asked of a representative sample of female voters, which allowed us to investigate the connection between gender linked fate and legal abortion attitudes in a large and representative group of women by marital status and race. In our methodological approach, we follow existing research of Stout, Kretschmer and Ruppanner [31] that used the 2012 ANES to show married white and Latina women report lower levels of gender linked fate than their non-married counterparts which helped explain their more conservative political attitudes. Using an equivalent methodological approach, we extend these relationships to support for legal abortion.

### The role of marriage and shifting perceptions of gender linked fate

Marriage is shown to alter women’s perceptions of the world that in turn shift their political and social attitudes, as described in detail previously [31]. Longitudinal analyses document that women become more conservative on gender-related issues and view themselves as having less in common with other women following marriage [48, 49]. For many women, marriage financially ties them to their husbands, and, by extension, they come to view changes in men’s economic and social standing as a threat to the family’s finances [50, 51, 52, 53, 54]. These experiences are counter to those of unmarried women who are more likely to be in poverty and work in insecure jobs [55]. As a result, unmarried women see their futures as more tightly linked to other women [31], in part, due to greater economic instability [56, 57]. Bolzendahl and Meyers [57] show that single women are more supportive of feminist issues than married women, with stronger effects among women who are more reliant on their own income. Married women, by contrast, are very unlikely to be sole breadwinners in their households [56] and, as a consequence, unlikely to see their futures as tied more explicitly to those of other women.

Marriage also traditionalizes domestic gender relations, with married women assuming a larger share of the housework even when their earnings exceed those of their husbands’ [58, 59]. Women who find themselves relying on their husbands’ income, tend to become less concerned about gender discrimination more broadly [60, 61]. Indeed, marriage appears to lessen women’s sense of ‘belonging’ to the broader female community, which is exhibited, in part, through married women’s lower support for policies to mitigate gender discrimination [60, 61]. Married women are also more likely to view changes in women’s social standing as coming at the expense of men’s social and economic status, and consequently, many view feminist politics as threatening to their own well-being because their husbands are threatened [62]. Collectively, these factors have been found to have a conservatizing effect, making married women less likely overall to support abortion rights [21].

Yet, absent from these discussions are the ways differing levels of gender linked fate among women with different marital status may condition these relationships. Since abortion is a procedure performed on women (and trans men) and thus is intrinsically connected to the futures of other women (and trans men), the traditionalizing effect of marriage on gender linked fate may be particularly pernicious for abortion support. Understanding the linkages between marital status and gender linked fate ought to provide a clearer picture of the conditions under which different women support legal abortion.

### Race, abortion and gender linked fate

Past research in the U.S. shows that gender linked fate varies across racial and ethnic lines with black women reporting the highest levels of gender consciousness and gender linked fate followed by Latinas, whites and Asian-Americans [31, 63, 64].

Further, existing research shows the relationship between marital status and gender linked fate is significant for white and Latina women, but not for black women [31]. For married white and Latina women, economic dependence on husbands tends to suppress perceptions of gender linked fate, but black women are more likely than women in other racial groups to be the primary breadwinner in their families [65]. Black women’s historical position as breadwinners makes them uniquely positioned to see their fate as tied to other women as black women’s experiences with both racial and gender inequality allow them to identify intersecting matrices of oppression and discrimination [45, 66]. This increases their capacity to empathize with other marginalized groups and to more closely identify with feminist political attitudes [67].

As described in detail previously [31] Latinas are more likely than other groups of women to have traditional breadwinner-homemaker divisions of labor, reinforcing wives’ financial dependence on their husbands [68, 69]. Latina women report spending the largest time in housework relative to black and white women and have the lowest rate of female breadwinners compared to all other racial groups, which may weaken their perceptions of gender linked fate [69, 70]. Latinas’ migration patterns may also weaken perceptions of gender linked fate, as many Latinas are first- and second-generation migrants who maintain the culture of their country of origin [71]. This includes strong support for “familism,” or the idea that the interests of the family as a whole take precedence over the interest of any single member of the family [68]. Since married Latina women are likely to be in breadwinning/homemaking families, support for familism likely shifts alliances to their husbands, thus weakening their perceptions of gender linked fate. Further, Latinos are more likely to be religious, and Protestant Latinos have been found to have particularly negative attitudes towards abortion [18, 19].

### Summary of expectations

Based on previous research, we expect women with lower levels of gender linked fate to report less support for legal abortion than women who see their fates as tied to other women. We expect these relationships to vary by race and marital status, consistent with previous research. Specifically, we expect married white and Latina women to report lower levels of abortion support than their non-married counterparts, in part, because of their lower levels of gender linked fate [31]. Our formal hypotheses are as follows:

*Hypothesis 1*: *Women with lower gender linked fate will report weaker support for legal abortion.*

*Hypothesis 2*: *Marital status will be linked to legal abortion attitudes among white and Latina women but not black women.*

*Hypothesis 3*: *Lower support for legal abortion among married white and Latina women will be, at least partially, explained by weaker perceptions of gender linked fate.*

### Data, measures and methods

We employed the 2012 American National Election Study (ANES) which provides a nationally representative survey of American attitudes on abortion and gender linked fate. The American National Election Survey is collected biennially to measure American’s political attitudes, voting preferences and demographics. The 2012 ANES is, to our knowledge, the only survey year in which gender linked fate was included in the survey instrument. Thus, we utilized the most up-to-date nationally representative data on this topic. We first tested for a gender gap in abortion attitudes, pooling the sample of men and women. Then, we restricted the sample to women because gender linked fate questions were presented *exclusively* to female respondents. This allowed us to determine whether gender linked fate helped explain abortion attitudes among women. The relatively large sample sizes provided by ANES also allowed us to estimate differences by race and marital status. Although we had adequate sample sizes for each racial group to estimate differences by marital status, we acknowledge that the number of white women in the sample is substantially larger than the numbers of black and Latina women, respectively.

For models assessing the gender gap in abortion support, our effective sample size was 2,235 men and 2,225 women (see S1 Table) Since gender linked fate was only asked of women, our effective sample was s 2,173 women who provided responses to the questions of interest (see S2 Table).

#### Abortion attitudes

Legal abortion attitudes served as our key dependent variable. We measured legal abortion support through a self-reported agreement with the following statement: “Do you favor, oppose, or neither favor nor oppose abortion being legal if the woman chooses to have one?”. Responses were measured on a 9-point scale ranging from (1) favor a great deal to (9) oppose a great deal. We reverse coded abortion attitudes so that higher values indicate greater support for legal abortion.

#### Key independent variables: Gender, gender linked fate, marital status and race

Initially, gender was used as a key explanatory factor to measure whether a gender gap in legal abortion support was evident in our sample. Those who self-identified as a man (value = 0) served as our reference group. Then, gender linked fate was used as a key mediator variable for a subsample of female respondents. Women were asked to report their levels of gender linked fate through a two-step process. First, they were asked: “Do you think that what happens generally to women in this country will have something to do with what happens in your life?”. If the respondent agreed to this question, they were then asked the following: “How much does what happens to others affect you?” Responses to this second question were measured on a three-point scale: (1) “a little”; (2) “some”; and (3) “a lot”. Higher values reflected stronger perceptions of gender linked fate.

We used these questions to construct a four-point gender linked fate measure. Respondents who answered “no” to the first question were given a score of 1, those who answered “a little” to the second question were given a score of 2, those who answered “some”–a score of 3, and those who answered “a lot” a score of 4. Our measure was consistent with the past measurement of linked fate for other groups (see [38, 64, 72, 73], and gender specifically [31].

Existing research shows gender linked fate varies by marital status and race [31]. We expected these differences to structure legal abortion support as well. To assess these hypotheses, we included measures of marital status and race. We compared single (i.e., never married) and divorced/separated women to their married counterparts. Women who stated that they were cohabiting with their partners (n = 134) were removed from the analysis. We excluded widows given their relatively small sample sizes across all racial groups (n = 176, n = 61 and n = 25 among white, black, and Latina women, respectively). We included self-reported race and compared white women to black and Latina women. The ANES does not have adequate sampling of Asian and bi-racial women and thus these women were excluded from our sample.

#### Attitudinal, political and sociodemographic controls

To isolate the effect of gender linked fate on legal abortion support, we included a range of potential confounders. Income and education have been consistently shown to be an important predictor of abortion support with more affluent and educated Americans more supportive of legal abortion. Thus, we included measures of the total household income and the amount of completed education in the model. Older Americans hold more conservative views on gender and politics and thus we included a measure of age in our models. We also controlled for having a child (eighteen years old or younger) at home, religiosity (measured by the frequency of church attendance on a scale from 1 –every week to 5 –never), political ideology (measured on a scale from 1 –extremely liberal to 7 –extremely conservative), and employment status (recoded as 1 –employed, 0 –other). Other’ included student, homemaker, retired, disabled, unemployed. Additional analyses with ‘homemaker’ as a separate category showed a similar pattern of results. We also investigated feminist attitudes as an alternative explanation for the tested link. Feminist attitudes were approximated by the following items: support for traditional gender roles (1 –better if man works and woman takes care of home, 2 –no difference, 3 –worse if man works and a woman takes care of the home), perceptions of gender discrimination (1 –not a problem; 5 –an extremely serious problem), and a feminist feeling thermometer (0–100). See S6 and S7 Tables for a summary of the results.

#### Analytical approach

**Fig 1:**
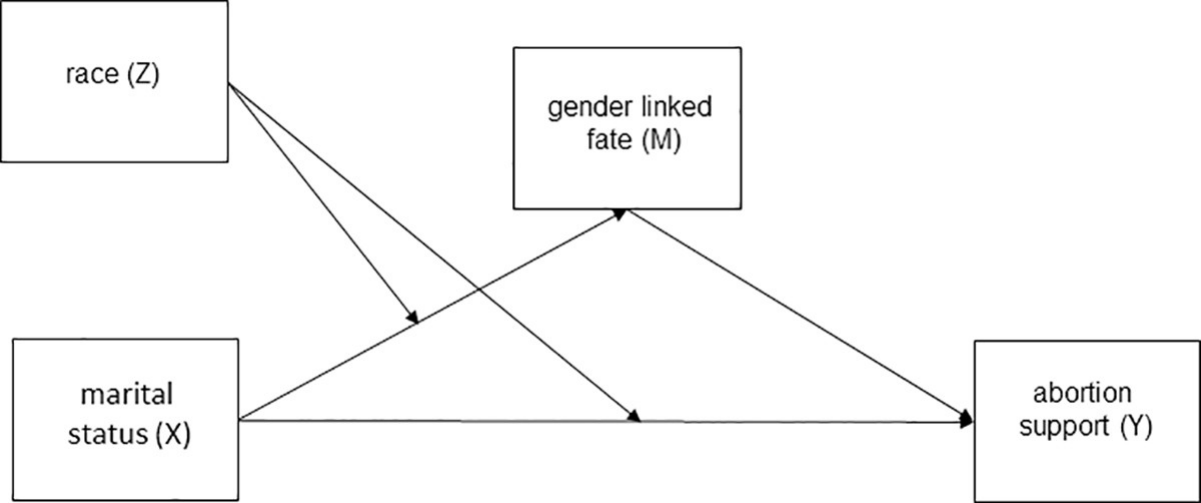
Moderated mediation model tested in the study. The model was estimated with ‘married’ as a reference group.

All analyses were computed using R (version 3.6.0, The R Foundation for Statistical Computing). Ordinal logistic regressions were calculated using package *ordinal*. Marginal means for gender linked fate and abortion support were calculated and plotted using package *emmeans* [74]. Mediation effects were calculated using natural effect models [75] included in package *medflex* [76], which allowed us to test a mediation model with an ordinal mediator, and to obtain estimates of indirect and direct effects of marital status conditional on race (Fig 1 shows a visual overview of our model).

## Results

Table 1 shows the results of a linear regression predicting legal abortion support among men and women, net of sociodemographic controls. Contrary to previous studies, our analysis revealed that respondent’s gender had a significant effect on support for legal abortion when controlling for other sociodemographic covariates.

**Table 1:** Linear regression predicting legal abortion support among men and women.

	*B*	*SE*	*p*	95% CI
Gender (1-female, 0-male)	0.36	0.09	<0.001	0.18, 0.54
Single	0.39	0.14	0.005	0.12, 0.66
Divorced/separated	0.13	0.13	0.334	-0.13, 0.39
Black	0.66	0.13	<0.001	0.40, 0.92
Latina	0.02	0.13	0.893	-0.24, 0.28
Age	0.01	0.01	0.003	0.01, 0.02
Education	0.26	0.04	<0.001	0.17, 0.34
Income	0.03	0.01	<0.001	0.02, 0.04
Employment status (1-employed, 0-other)	0.26	0.10	0.011	0.06, 0.46
Have children (eighteen or younger) at home	-0.05	0.12	0.668	-0.28, 0.18
Religiosity (frequency of church attendance; 1-every week, 5-never)	0.67	0.03	<0.001	0.61, 0.73
Ideology (1-liberal, 7-conservative)	-0.76	0.03	<0.001	-0.83, -0.70

N = 3,785 (675 observations were deleted due to missing data); CI–Confidence Intervals.

**Fig 2:**
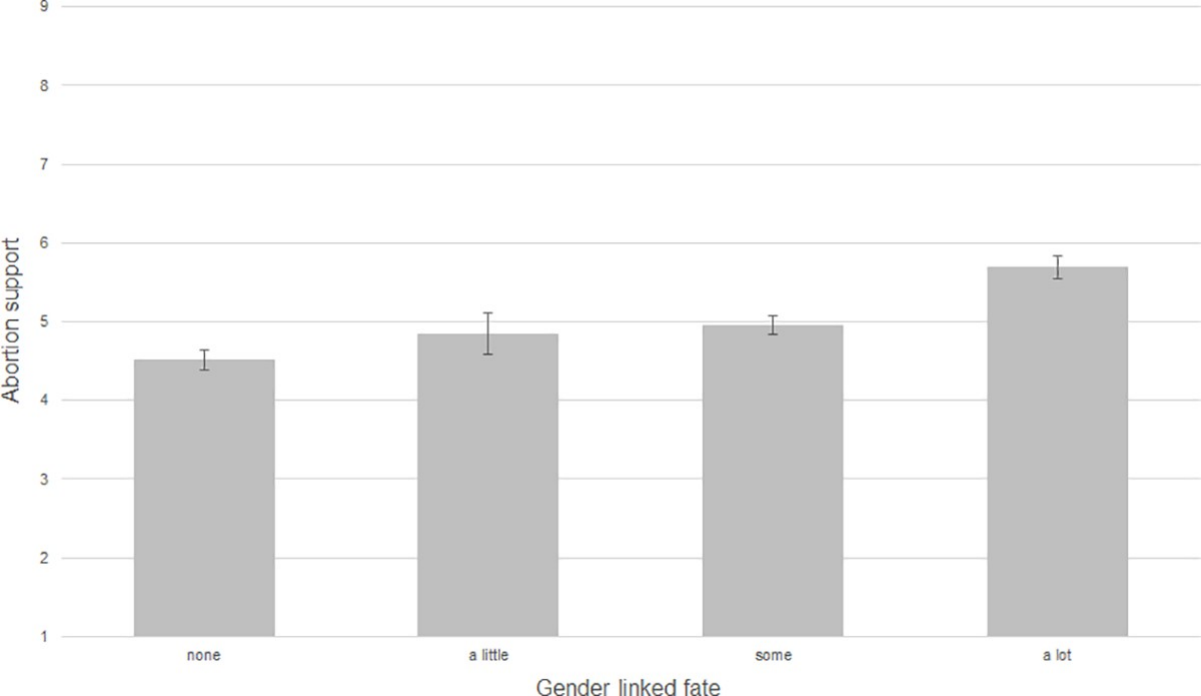
Gender linked fate and legal abortion support. Source: 2012 American National Election Study. rτ = 0.10, *p* < 0.001.

Next, we assessed the relationship between gender linked fate and abortion attitudes for women. Fig 2 provides a visual overview of the relationship between gender linked fate and support for abortion. Our first hypothesis predicted that women with lower levels of gender linked fate would report weaker support for abortion. Fig 2 confirms this expectation–women who viewed their futures as less tied to other women did report weaker support for abortion (rτ = 0.10, *p* < 0.001).

**Fig 3:**
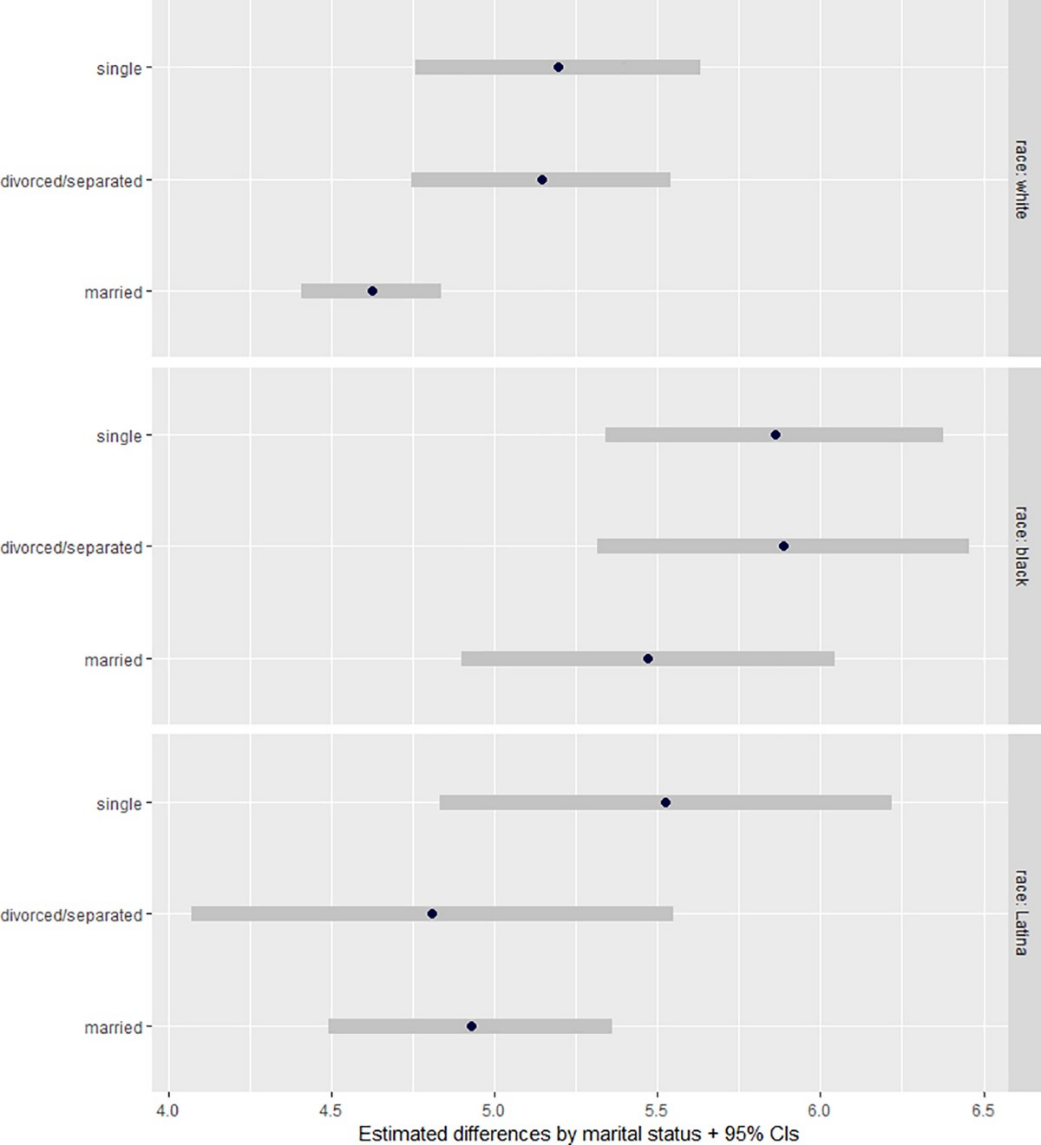
Predicted levels of legal abortion support by marital status and racial/ethnic group. Source: 2012 American National Election Study. CI = Confidence Interval. white: n = 221 single, n = 237 divorced/separated, n = 881 married; black: n = 189 single, n = 140 divorced/separated, n = 132 married; Latina: n = 94 single, n = 78 divorced/separated; n = 201 married. Effects were adjusted for age, income, employment status, education, having children (eighteen or younger) at home, religiosity (frequency of church attendance) and political ideology.

Guided by previous research, we expected that marital status would be linked to legal abortion support among white and Latina women, but not among black women. Results presented in Fig 3 lend partial support for this claim (detailed results can be found in S3 and S4 Tables). White married women were less supportive of abortion than their single or divorced counterparts. By contrast, black and Latina women’s level of abortion support did not differ by marital status, countering our expectations that Latina women’s gender linked fate would be structured similarly to white women’s.

Finally, we expected that the difference in abortion support by marital status for white and Latina women would be explained by their lower levels of gender linked fate. Table 2 explores these relationships and lends partial support for this hypothesis (full details of the analysis can be found in S3–S6 Tables). Married white women’s lower levels of gender linked fate, relative to single white women, explained 16.3% of the relationship between marriage and abortion attitudes. A similar relationship was found when comparing married white women to their divorced/separated counterparts. Lower levels of gender linked fate among married white women, compared to divorced/separated white women, explained 14.5% of the effect of marriage on abortion. The relationships between gender linked fate, marriage and abortion support were significant only among white women indicating that marriage plays a distinct role in gender linked fate attitudes for this group.

**Table 2:** Mediation analysis predicting legal abortion support via gender linked fate.

	White	Black	Latina
	b [95% CI]	b [95% CI]	b [95% CI]
**Single vs married**			
Indirect effect	0.09* [0.02, 0.18]	-0.01 [-0.15, 0.17]	0.04 [-0.21, 0.29]
Direct effect	0.48 [-0.01, 0.97]	0.40 [-0.47, 1.21]	0.56 [-0.40, 1.49]
Total effect	0.57* [0.11, 1.07]	0.39 [-0.49, 1.23]	0.60 [-0.33, 1.51]
Prop. mediated	16.3%	*ns*	*ns*
**Divorced/separated vs married**			
Indirect effect	0.08* [0.02, 0.15]	0.03 [-0.12, 0.17]	0.07 [-0.08, 0.27]
Direct effect	0.44 [-0.05, 0.90]	0.39 [-0.48, 1.19]	-0.19 [-1.08, 0.78]
Total effect	0.52* [0.04, 0.99]	0.42 [-0.46, 1.25]	-0.12 [-1.03, 0.83]
Prop. mediated	14.5%	*ns*	*ns*

* *p*<0.05, **p<0.01; 95%CI–bootstrap percentile confidence intervals based on 1,000 bootstrap samples; Effects were adjusted for age, income, employment status, education, having children (eighteen or younger) at home, religiosity (frequency of church attendance) and political ideology.

Additionally, we conducted several sensitivity analyses to confirm that the observed link between marital status and abortion attitudes is attributable to gender linked fate, not feminist attitudes (see S8 Table). None of the alternative mediators considered in the analysis explained a significant proportion of variance. Additionally, we tested whether the observed relationships might vary with the respondent’s age and employment status (see S9–S11 Tables). The analysis indicated that, among white respondents, marital status had a stronger effect on gender linked fate among younger respondents (<45) but did not vary with employment status. A similar age trend could be observed for black and Latina respondents. However, these results were not significant and should be treated with caution due to small numbers, particularly of young (<45) divorced/separated women. For similar reasons, the results of the moderated mediation analysis presented in S1 Table should be also only considered as tentative.

## Discussion

Abortion is an increasingly important political issue in the United States. The recent passage of an abortion ban in Alabama and abortion restrictions in other states are a clear indication that legal abortion is an increasingly important political issue with serious implications for women. Here, we investigate how gender linked fate, or the perception that a woman’s future is intrinsically tied to other women, is associated with support for abortion. Because abortion is intrinsically tied to women’s bodies, perceptions of gender linked fate are an important explanatory mechanism. Research on public attitudes has consistently found stark divides in support for abortion across religiousness, race, region, and political ideology [1, 3, 7, 8]. Yet, the link to gender linked fate by race and marital status is conspicuously absent–this study fills that void. Our study is important as women, especially white married women, are playing an increasingly critical role in American politics, demonstrated by the fracture of suburban white women from the Republican party and its impact on the 2018 midterm election. Women make up the majority of the population, have high voting participation rates, and, on average tend to vote in more liberal ways [77]. Given the increasing salience of abortion in every level of politics and the rising influence of women, investigation of drivers of abortion attitudes among women deserves greater attention. Here, we untangled one underlying explanation of women’s attitudes toward legal abortion by paying attention to differences in gender linked fate by marital status.

We find that women are, on average more supportive of abortion than men, a finding that counters previous research that fails to document a gender gap [33–36]. We then find that women with weaker perceptions of gender linked fate report lower levels of legal abortion support. This relationship is meaningful for academics to understand gender linked fate as an explanatory mechanism of attitudes, and politicians and advocacy groups to develop more effective political messaging. Here, we show that among the female population, gender linked fate is an important precursor for abortion support. As a consequence, shifting perceptions of gender linked fate may impact attitudes towards legal abortion.

After first identifying this generalized trend, we then identify racial patterns of legal abortion support by marital status. As predicted, white married women report lower levels of legal abortion support than their single counterparts. By contrast, black and Latina women reported equivalent levels of legal abortion support across marital statuses. Our results indicate that marriage plays a distinct role in structuring legal abortion attitudes for white women. This finding is, perhaps, not surprising in light of the literature on racial linked fate whereby people of color see their futures as more intrinsically linked compared to white respondents [38, 45, 47, 66]. Existing scholarship shows black women are uniquely positioned to identify compounding matrices of oppression by race and gender, seeing their futures as more tightly linked to blacks *and* other women [45, 66]. These identities appear to extend to abortion attitudes as well in that, for women of color, marriage does not lower support for legal abortion nor do their lower levels of gender linked fate explain the relationship between marriage and legal abortion attitudes. Again, this appears to be a relationship distinct from white women’s experiences.

Our findings parallel existing scholarship that shows marriage is traditionalizing [48, 49] and emerging literature that identifies an underlying causal mechanism–gender linked fate [31]. In this regard, marriage appears to have a particular traditionalizing impact for white women that is not evident for other racial groups identifying an interesting pathway for future longitudinal research.

Notably, we estimate these relationships net of a range of sociodemographic controls including income, education, and employment status. We also compare alternative explanations of the observed link looking at different proxies of feminist attitudes. Our results indicate that gender linked fate is a robust and theoretically distinct concept that structures married white women’s attitudes.

## Limitations and future directions

The 2012 ANES had several limitations in regards to our study. First, the gender linked fate question was only presented to female, not male respondents. Although, as we have argued, abortion has a unique meaning for women to the extent that it is intrinsically tied to their bodies, we cannot exclude that men’s level of connection to other women (or to other men) could explain their support or opposition to legal abortion. This points to a data gap that could be explored in subsequent studies. Another limitation is that, while ANES provides sufficient numbers of women by race (for white, black and Latina respondents), and marital status (for married, divorced/separated and single women) to allow for comparisons, the majority of the sample is white. It is feasible that a larger sample including more black and Latina women may have captured marriage status differences in gender linked fate and abortion attitudes across these groups. Future studies should test whether the unique effect we find for white women can be observed in other minorities. Finally, given that the focus of ANES is on political attitudes more broadly, the questionnaire did not include other possible moderators of abortion attitudes and gender linked fate, such as access to contraception or length of marriage.

We believe that our study provides a promising avenue for future research, including longitudinal designs which would allow for testing whether marriage traditionalizes attitudes or whether more traditional men and women are more likely to marry, and to uncover a “dose-effect” of marriage, that is the extent to which duration of marriage structures perceptions of gender linked fate and abortion attitudes. Experimental methods could further shed light on the causal relationship between marriage, gender linked fate and abortion attitudes. Future studies could also explore how contraception use and past abortion experiences structure abortion support. Finally, future studies could also aim to uncover the link between gender linked fate and group consciousness paying careful attention to men as well as women.

## Conclusion

Our results from a nationally-representative sample of American women indicate that abortion attitudes, similar to general political conservativism, vary by marital status and perceptions of gender linked fate. Crucially, we show that married white women see their futures and fates as less tied to other women than non-married white women and, as a consequence, are less supportive of legal abortion. These findings are important in the context of changing political alliances in the post-Trump era whereby many suburban educated white women split from the Republican party in the 2018 midterms. Whether this fissure is temporary or long-lasting is an empirical question that cannot yet be answered, but our results suggest that abortion support, like general political conservatism, depends on race, marital status and perceptions of gender linked fate. The passage of restrictive abortion laws in many states with high concentrations of white married women suggests a need to refine this area of inquiry and further exploration of the underlying mechanisms of abortion attitudes.

## Supporting information

S1 Table Sample profile. (PDF)

S2 Table Sample profile of women included in the analysis of gender linked fate. (PDF)

S3 Table Linear regression predicting abortion support among women. (PDF)

S4 Table Conditional effects of marital status on abortion support, by race. (PDF)

S5 Table Ordered logistic regression predicting gender linked fate. (PDF)

S6 Table Conditional effects of marital status on gender linked fate, by race. (PDF)

S7 Table Descriptive statistics for alternative mediators considered in the analysis. (PDF)

S8 Table Alternative mediation models explaining the marital status–abortion support link. (PDF)

S9 Table Conditional effects of marital status on gender linked fate, by race and employment status. (PDF)

S10 Table Conditional effects of marital status on gender linked fate, by race and age. (PDF)

S11 Table Mediation models by age and employment status. (PDF)
