# Supplementary material for: Gender linked fate explains lower legal abortion support among white married women
Source: PLoS One. 2019 Oct 10;14(10):e0223271. doi: 10.1371/journal.pone.0223271 (PMC6786754; doi:10.1371/journal.pone.0223271)
Supplement: S1 Table — (PDF) [file pone.0223271.s001.pdf]

S1 Table. Sample Profile. *N* = 4,460; Numbers might not sum up to 100% due to missing data; *'Other' included: student, unemployed, homemaker, disabled, retired.

	Female (n = 2,225)		*Male (n = 2,235)*	
	N	*%*	*N*	*%*
Married	1,248	56.1	1,351	60.4
Single	513	23.1	534	15.7
Divorced/separated	464	20.9	350	23.9
White	1,371	61.6	1,497	67.0
Black	470	21.1	358	16.0
Latina/Latino	384	17.3	380	17.0
Employed	1,100	49.4	1,276	57.1
Other*	1,123	50.5	955	42.7
Have children (eighteen or younger) at home	834	37.5	643	28.8
No children	1,390	62.5	1,590	71.1
	M	*SD*	*M*	*SD*
Age	48.10	15.68	49.88	16.21
Income (1-under $5,000, 28-$250,000 or more)	13.58	8.03	14.96	8.12
Education (1-less than high school credential, 5-graduate degree)	2.98	1.15	3.06	1.16
Religiosity (frequency of church attendance; 1-every week, 5-never)	3.20	1.63	3.48	1.62
Ideology (1-liberal, 7-conservative)	4.09	1.49	4.26	1.46
Abortion support (1-oppose a great deal, 9-favor a great deal)	4.98	3.41	4.79	3.27
