# Supplementary material for: Gender linked fate explains lower legal abortion support among white married women
Source: PLoS One. 2019 Oct 10;14(10):e0223271. doi: 10.1371/journal.pone.0223271 (PMC6786754; doi:10.1371/journal.pone.0223271)
Supplement: S2 Table — (PDF) [file pone.0223271.s002.pdf]

**S2 Table. Sample Profile of Women Included in the Analysis of Gender Linked Fate.** *N* = 2,173*;* Numbers might not sum up to 100% due to missing data; *'Other' included: student, unemployed, homemaker, disabled, retired; Analysis with 'homemaker' as a separate category did not alter the results.

| | *White (n = 1,339)* | | *Black (n = 461)* | | *Latina (n = 373)* | |
|---|---|---|---|---|---|---|
| | *N* | *%* | *N* | *%* | *N* | *%* |
| Married | 881 | 65.8 | 132 | 28.6 | 201 | 53.9 |
| Single | 221 | 16.5 | 189 | 41.0 | 94 | 25.2 |
| Divorced/separated | 237 | 17.7 | 140 | 30.4 | 78 | 20.9 |
| Employed | 664 | 49.4 | 231 | 50.1 | 182 | 48.8 |
| Other* | 674 | 50.3 | 230 | 49.9 | 190 | 50.9 |
| Have children (eighteen or younger) at home | 406 | 69.6 | 252 | 54.7 | 179 | 48.0 |
| No children | 932 | 30.3 | 209 | 45.3 | 194 | 52.0 |
| Gender linked fate (none) | 399 | 29.8 | 153 | 33.2 | 136 | 36.5 |
| Gender linked fate (a little) | 89 | 6.6 | 22 | 4.8 | 40 | 10.7 |
| Gender linked fate (some) | 532 | 39.7 | 165 | 35.8 | 107 | 28.7 |
| Gender linked fate (a lot) | 319 | 23.8 | 121 | 26.2 | 90 | 24.1 |
| | *M* | *SD* | *M* | *SD* | *M* | *SD* |
| Age | 50.47 | 15.48 | 45.59 | 14.71 | 48.88 | 15.62 |
| Education (1-less than high school credential, 5-graduate degree) | 3.16 | 1.12 | 2.77 | 1.10 | 2.61 | 1.13 |
| Income (1-under $5,000, 28-$250,000 or more) | 15.35 | 7.90 | 10.03 | 7.32 | 11.06 | 7.28 |
| Religiosity (frequency of church attendance; 1-every week, 5-never) | 3.35 | 1.65 | 2.81 | 1.52 | 3.14 | 1.59 |
| Ideology (1-liberal, 7-conservative) | 4.26 | 1.51 | 3.63 | 1.34 | 3.95 | 1.42 |
| Abortion support (1-oppose a great deal, 9-favor a great deal) | 4.84 | 3.47 | 5.58 | 3.22 | 4.72 | 3.32 |
