# Supplementary material for: Gender linked fate explains lower legal abortion support among white married women
Source: PLoS One. 2019 Oct 10;14(10):e0223271. doi: 10.1371/journal.pone.0223271 (PMC6786754; doi:10.1371/journal.pone.0223271)
Supplement: S3 Table — (PDF) [file pone.0223271.s003.pdf]

S3 Table. Linear Regression Predicting Abortion Support Among Women. *N* = 1,803 (370 observations were deleted due to missing data); CI – Confidence Intervals.

X → Y	*B*	*SE*	*p*	95% CI
Single	0.53	0.20	0.010	0.13, 0.92
Divorced/separated	0.40	0.19	0.032	0.03, 0.77
Black	0.77	0.20	<0.001	0.38, 1.15
Latina	0.19	0.19	0.325	-0.19, 0.57
Age	0.01	0.01	0.033	0.01, 0.02
Education	0.16	0.07	0.013	0.03, 0.29
Income	0.05	0.01	<0.001	0.03, 0.07
Employment status (1-employed, 0-other)	0.43	0.14	0.003	0.15, 0.71
Have children (eighteen or younger) at home	0.05	0.16	0.784	-0.28, 0.37
Religiosity (frequency of church attendance; 1-every week, 5 - never)	0.64	0.04	<0.001	0.56, 0.73
Ideology (1- liberal, 7 - conservative)	-0.80	0.05	<0.001	-0.90, -0.71

X^Z → Y	*B*	*SE*	*p*	95% CI
Single	0.57	0.26	0.026	0.07, 1.07
Divorced/separated	0.52	0.23	0.025	0.06, 0.98
Black	0.85	0.31	0.007	0.24, 1.46
Latina	0.30	0.25	0.221	-0.18, 0.79
Single x Black	-0.18	0.45	0.685	-1.06, 0.70
Single x Latina	0.03	0.46	0.954	-0.88, 0.93
Divorced/separated x Black	-0.10	0.46	0.820	-1.01, 0.80
Divorced/separated x Latina	-0.64	0.49	0.189	-1.59, 0.32
Age	0.01	0.01	0.003	0.01, 0.02
Education	0.16	0.07	0.015	0.03, 0.29
Income	0.05	0.01	<0.001	0.03, 0.07
Employment status (1-employed, 0-other)	0.42	0.14	0.003	0.14, 0.70
Have children (eighteen or younger) at home	0.06	0.17	0.735	-0.27, 0.38
Religiosity (frequency of church attendance; 1-every week, 5 - never)	0.64	0.04	<0.001	0.55, 0.73
Ideology (1- liberal, 7 - conservative)	-0.80	0.05	<0.001	-0.90, -0.70

X^Z (M) → Y	*B*	*SE*	*p*	95% CI
Single	0.51	0.26	0.049	0.01, 1.01
Divorced/separated	0.46	0.23	0.050	-0.00, 0.91
Black	0.85	0.31	0.006	0.24, 1.46
Latina	0.33	0.25	0.189	-0.16, 0.81
Single x Black	-0.12	0.45	0.787	-1.00, 0.76
Single x Latina	0.01	0.46	0.977	-0.89, 0.92
Divorced/separated x Black	-0.08	0.46	0.855	-0.99, 0.82
Divorced/separated x Latina	-0.62	0.49	0.201	-1.58, 0.33
Age	0.01	<0.01	0.016	0.01, 0.02
Education	0.14	0.07	0.036	0.01, 0.27
Income	0.05	0.01	<0.001	0.03, 0.07
Employment status (1-employed, 0-other)	0.42	0.14	0.003	0.14, 0.70
Have children (eighteen or younger) at home	0.05	0.17	0.746	-0.27, 0.38
Religiosity (frequency of church attendance; 1-every week, 5 - never)	0.65	0.04	<0.001	0.56, 0.73
Ideology (1- liberal, 7 - conservative)	-0.78	0.05	<0.001	-0.89, -0.68
Gender linked fate (a little)	0.06	0.27	0.822	-0.47, 0.60
Gender linked fate (some)	0.12	0.17	0.455	-0.21, 0.45
Gender linked fate (a lot)	0.49	0.19	0.011	0.11, 0.86
