# Supplementary material for: Gender linked fate explains lower legal abortion support among white married women
Source: PLoS One. 2019 Oct 10;14(10):e0223271. doi: 10.1371/journal.pone.0223271 (PMC6786754; doi:10.1371/journal.pone.0223271)
Supplement: S4 Table — (PDF) [file pone.0223271.s004.pdf]

S4 Table. Conditional Effects of Marital Status on Abortion Support, by Race. *N* = 1,792; CI – Confidence Intervals; Effects were adjusted for age, income, employment status, education, having children (eighteen or younger) at home, religiosity (frequency of church attendance; 1- every week, 5 - never), political ideology (1 – extremely liberal, 7 – extremely conservative), and traditional gender roles.

X^Z → Y	*B*	*SE*	*p*	95% CI
**White**				
Single	0.57	0.26	0.049	0.01, 1.14
Divorced/separated	0.52	0.23	0.048	0.01, 1.04
**Black**				
Single	0.39	0.39	0.605	-0.48, 1.26
Divorced/separated	0.42	0.41	0.493	-0.49, 1.32
**Latina**				
Single	0.60	0.42	0.264	-0.33, 1.52
Divorced/separated	-0.12	0.43	0.934	-1.08, 0.85
