# Supplementary material for: Gender linked fate explains lower legal abortion support among white married women
Source: PLoS One. 2019 Oct 10;14(10):e0223271. doi: 10.1371/journal.pone.0223271 (PMC6786754; doi:10.1371/journal.pone.0223271)
Supplement: S5 Table — (PDF) [file pone.0223271.s005.pdf]

S5 Table. Ordered Logistic Regression Predicting Gender Linked Fate. *N* = 1,792; CI – Confidence Intervals.

X → M	*B*	*SE*	*p*	95% CI
Single	0.31	0.08	<0.001	0.16, 0.47
Divorced/separated	0.38	0.07	<0.001	0.23, 0.53
Black	-0.15	0.08	0.044	-0.31, -0.01
Latina	-0.15	0.08	0.055	-0.30, 0.00
Age	-0.01	<0.01	<0.001	-0.01, -0.01
Education	0.18	0.03	<0.001	0.12, 0.23
Income	0.01	<0.01	0.094	-0.01, 0.01
Employment status (1-employed, 0-other)	-0.01	0.06	0.875	-0.12, 0.10
Have children (eighteen or younger) at home	-0.04	0.06	0.559	-0.16, 0.09
Religiosity (frequency of church attendance; 1- every week, 5 - never)	-0.04	0.01	0.019	-0.07, -0.01
Ideology (1- liberal, 7 - conservative)	-0.11	0.02	<0.001	-0.14, -0.07
Cut 1	-0.90	0.21		-1.31, -0.50
Cut 2	-0.69	0.21		-1.10, -0.28
Cut 3	0.40	0.21		-0.01, 0.81

X^Z → M	*B*	*SE*	*p*	95% CI
Single	0.36	0.10	<0.001	0.16, 0.56
Divorced/separated	0.39	0.09	<0.001	0.21, 0.57
Black	-0.02	0.12	0.864	-0.26, 0.22
Latina	-0.18	0.10	0.064	-0.38, 0.01
Single x Black	-0.28	0.18	0.109	-0.63, 0.06
Single x Latina	0.15	0.18	0.424	-0.21, 0.51
Divorced/separated x Black	-0.11	0.18	0.549	-0.47, 0.25
Divorced/separated x Latina	-0.02	0.19	0.931	-0.39, 0.36
Age	-0.01	<0.01	<0.001	-0.01, -0.01
Education	0.17	0.03	<0.001	0.12, 0.22
Income	0.01	<0.01	0.095	-0.01, 0.01
Employment status (1-employed, 0-other)	-0.01	0.06	0.877	-0.12, 0.10
Have children (eighteen or younger) at home	-0.03	0.07	0.676	-0.15, 0.10
Religiosity (frequency of church attendance; 1- every week, 5 - never)	-0.04	0.02	0.023	-0.07, -0.01
Ideology (1- liberal, 7 - conservative)	-0.10	0.02	<0.001	-0.14, -0.07
Cut 1	-0.87	0.21		-1.28, -0.46
Cut 2	-0.65	0.21		-1.07, -0.24
Cut 3	0.44	0.21		-0.02, 0.84
