# Supplementary material for: Gender linked fate explains lower legal abortion support among white married women
Source: PLoS One. 2019 Oct 10;14(10):e0223271. doi: 10.1371/journal.pone.0223271 (PMC6786754; doi:10.1371/journal.pone.0223271)
Supplement: S6 Table — (PDF) [file pone.0223271.s006.pdf]

S6 Table. Conditional Effects of Marital Status on Gender Linked Fate, by Race. *N* = 1,792; CI – Confidence Intervals; Effects were adjusted for age, income, employment status, education, having children (eighteen or younger) at home, religiosity (frequency of church attendance; 1- every week, 5 - never), and political ideology (1 – extremely liberal, 7 – extremely conservative).

X^Z → M	*B*	*SE*	*p*	95% CI
**White**				
Single	0.36	0.10	<0.001	0.13, 0.58
Divorced/separated	0.39	0.09	<0.001	0.19, 0.60
**Black**				
Single	0.07	0.15	0.834	-0.27, 0.41
Divorced/separated	0.28	0.16	0.140	-0.07, 0.64
**Latina**				
Single	0.51	0.17	0.005	0.14, 0.87
Divorced/separated	0.38	0.17	0.053	-0.01, 0.75
