# Supplementary material for: Gender linked fate explains lower legal abortion support among white married women
Source: PLoS One. 2019 Oct 10;14(10):e0223271. doi: 10.1371/journal.pone.0223271 (PMC6786754; doi:10.1371/journal.pone.0223271)
Supplement: S7 Table — (PDF) [file pone.0223271.s007.pdf]

S7 Table. Descriptive Statistics for Alternative Mediators Considered in the Analysis. *N* = 2,173*;* Numbers might not sum up to 100% due to missing data.

	White (n = 1,339)		*Black (n = 461)*		*Latina (n = 373)*	
	N	*%*	*N*	*%*	*N*	*%*
Traditional gender roles (better if man works)	602	45.0	168	36.4	183	49.1
Traditional gender roles (no difference)	685	51.2	260	56.4	166	44.5
Traditional gender roles (worse)	43	3.2	28	6.1	23	6.2
	M	*SD*	*M*	*SD*	*M*	*SD*
Discrimination (1-not a problem, 5-an extremely serious problem)	2.74	0.88	3.26	0.97	2.90	1.02
Feminist feeling thermometer (0-100)	51.71	23.23	60.30	23.28	57.96	25.87
