# Supplementary material for: Gender linked fate explains lower legal abortion support among white married women
Source: PLoS One. 2019 Oct 10;14(10):e0223271. doi: 10.1371/journal.pone.0223271 (PMC6786754; doi:10.1371/journal.pone.0223271)
Supplement: S8 Table — (PDF) [file pone.0223271.s008.pdf]

S8 Table. Alternative Mediation Models Explaining the Marital Status – Abortion Support Link. *$p<0.05$, **$p<0.01$; 95%CI – bootstrap percentile confidence intervals based on 1,000 bootstrap samples; *Note*. Effects were adjusted for age, income, employment status, education, having children (eighteen or younger) at home, religiosity (frequency of church attendance), and political ideology.

Indirect effects	*White*	*Black*	*Latina*
	b [95% CI]	b [95% CI]	b [95% CI]
**Single vs married**			
Gender linked fate	0.09* [0.02, 0.18]	-0.01 [-0.15, 0.17]	0.04 [-0.21, 0.29]
Discrimination	0.03 [-0.06, 0.12]	-0.06 [-0.21, 0.06]	-0.12 [-0.33, 0.06]
Traditional gender roles	0.03 [-0.03, 0.10]	-0.08 [-0.31, 0.10]	0.13 [-0.05, 0.35]
Feminist feeling thermometer	0.06 [-0.03, 0.14]	-0.11 [-0.29, 0.02]	-0.01 [-0.01, 0.11]
**Divorced/separated vs married**			
Gender linked fate	0.08* [0.02, 0.15]	0.03 [-0.12, 0.17]	0.07 [-0.08, 0.27]
Discrimination	0.03 [-0.03, 0.11]	-0.06 [-0.20, 0.03]	-0.07 [-0.24, 0.06]
Traditional gender roles	0.02 [-0.03, 0.07]	0.07 [-0.05, 0.20]	-0.01 [-0.12, 0.15]
Feminist feeling thermometer	0.06 [-0.01, 0.13]	-0.01 [-0.13, 0.11]	0.02 [-0.12, 0.15]
