# Supplementary material for: Gender linked fate explains lower legal abortion support among white married women
Source: PLoS One. 2019 Oct 10;14(10):e0223271. doi: 10.1371/journal.pone.0223271 (PMC6786754; doi:10.1371/journal.pone.0223271)
Supplement: S9 Table — (PDF) [file pone.0223271.s009.pdf]

S9 Table. Conditional Effects of Marital Status on Gender Linked Fate, by Race and Employment Status. *N* = 1,792; CI – Confidence Intervals; Effects were adjusted for age (employment model only), income, employment status (age model only), education, having children (eighteen or younger) at home, religiosity (frequency of church attendance; 1- every week, 5 - never), and political ideology (1 – extremely liberal, 7 – extremely conservative); Due to relatively low numbers in particular subgroups (e.g., *n* = 37 for divorced/separated and employed Latinas), results should be treated with caution and the analysis might not have had enough statistical power to detect significant effects.

	B	*SE*	*p*	95% CI
**White (employed)**				
Single	0.33	0.13	0.016	0.05, 0.61
Divorced/separated	0.41	0.12	0.002	0.13, 0.68
**White (other)**				
Single	0.37	0.15	0.026	0.04, 0.71
Divorced/separated	0.37	0.13	0.010	0.08, 0.70
**Black (employed)**				
Single	0.23	0.22	0.462	-0.25, 0.71
Divorced/separated	0.33	0.23	0.267	-0.18, 0.84
**Black (other)**				
Single	-0.08	0.21	0.891	-0.55, 0.40
Divorced/separated	0.25	0.22	0.428	-0.24, 0.74
**Latina (employed)**				
Single	0.61	0.22	0.011	0.12, 1.01
Divorced/separated	0.46	0.24	0.103	-0.07, 0.99
**Latina (other)**				
Single	0.42	0.24	0.160	-0.13, 0.96
Divorced/separated	0.30	0.24	0.354	-0.23, 0.84
