# Supplementary material for: Gender linked fate explains lower legal abortion support among white married women
Source: PLoS One. 2019 Oct 10;14(10):e0223271. doi: 10.1371/journal.pone.0223271 (PMC6786754; doi:10.1371/journal.pone.0223271)
Supplement: S10 Table — (PDF) [file pone.0223271.s010.pdf]

S10 Table. Conditional Effects of Marital Status on Gender Linked Fate, by Race and Age. *N* = 1,792; CI – Confidence Intervals; Effects were adjusted for age (employment model only), income, employment status (age model only), education, having children (eighteen or younger) at home, religiosity (frequency of church attendance; 1- every week, 5 - never), and political ideology (1 – extremely liberal, 7 – extremely conservative); Due to relatively small numbers of women in particular subgroups (e.g., *n* = 11 for the single Latinas 45+; *n* = 27 for divorced/separated Latinas and Black women under 45), results should be treated with caution and the analysis might not have enough statistical power to detect significant effects.

	B	*SE*	*p*	95% CI
**White <45**				
Single	0.65	0.14	<0.001	0.34, 0.95
Divorced/separated	0.54	0.21	0.020	0.07, 1.00
**White 45+**				
Single	0.27	0.14	0.108	-0.05, 0.58
Divorced/separated	0.32	0.10	0.003	0.09, 0.54
**Black <45**				
Single	0.30	0.21	0.271	-0.02, 0.78
Divorced/separated	0.51	0.30	0.155	-0.15, 1.17
**Black 45+**				
Single	-0.12	0.24	0.823	-0.65, 0.41
Divorced/separated	0.18	0.19	0.560	-0.25, 0.61
**Latina <45**				
Single	0.65	0.19	0.001	0.22, 1.08
Divorced/separated	0.64	0.29	0.049	0.001, 1.27
**Latina 45+**				
Single	0.50	0.38	0.328	-0.35, 1.34
Divorced/separated	0.23	0.22	0.461	-0.25, 0.72
