# Supplementary material for: Gender linked fate explains lower legal abortion support among white married women
Source: PLoS One. 2019 Oct 10;14(10):e0223271. doi: 10.1371/journal.pone.0223271 (PMC6786754; doi:10.1371/journal.pone.0223271)
Supplement: S11 Table — (PDF) [file pone.0223271.s011.pdf]

S11 Table. Mediation Models by Age and Employment Status. * $p<0.05$; 95% CI – bootstrap percentile confidence intervals based on 1,000 bootstrap samples; Effects were adjusted for age (employment status model only), income, employment status (age model only), education, having children (eighteen or younger) at home, religiosity (frequency of church attendance), and political ideology. Due to problems with model convergence, analyses were performed with gender linked fate recoded into 3-categories (none, a little/some, a lot) and treated as a continuous variable. Due to relatively small numbers of women in particular subgroups (e.g., n = 37 for divorced/separated and employed Latinas; n = 11 for the single Latinas 45+; n = 27 for divorced/separated Latinas and Black women under 45), results should be treated with caution, and the analysis might not have enough statistical power to detect significant effects.

Indirect effects	*White*	*Black*	*Latina*
	b [95% CI]	b [95% CI]	b [95% CI]
**Single vs married**			
employed	0.07 [-0.01, 0.19]	0.01 [-0.11, 0.12]	0.12 [-0.24, 0.54]
other	0.05 [-0.04, 0.19]	-0.02 [-0.18, 0.09]	0.01 [-0.30, 0.37]
<45	0.03 [-0.12, 0.19]	0.05 [-0.10, 0.26]	0.17 [-0.08, 0.51]
45+	0.06 [-0.01, 0.17]	0.02 [-0.16, 0.18]	-0.02 [-0.43, 0.32]
**Divorced/separated vs married**			
employed	0.07 [0.00, 0.17]	0.01 [-0.12, 0.16]	0.09 [-0.14, 0.39]
other	0.05 [-0.02, 0.16]	0.03 [-0.11, 0.18]	0.02 [-0.12, 0.20]
<45	0.02 [-0.10, 0.16]	0.11 [-0.10, 0.44]	0.16 [-0.09, 0.56]
45+	0.08* [0.02, 0.17]	0.01 [-0.10, 0.10]	-0.07 [-0.21, 0.16]
